# Pilot Study on Clinical Effectiveness of Autofluorescence Imaging for Early Gastric Cancer Diagnosis by Less Experienced Endoscopists

**DOI:** 10.1155/2011/419136

**Published:** 2011-07-21

**Authors:** Kazuhiro Tada, Ichiro Oda, Chizu Yokoi, Tomoyasu Taniguchi, Taku Sakamoto, Haruhisa Suzuki, Satoru Nonaka, Shigetaka Yoshinaga, Yutaka Saito, Takuji Gotoda

**Affiliations:** ^1^Endoscopy Division, National Cancer Center Hospital, 5-1-1 Tsukiji, Chuo-ku, Tokyo 104-0045, Japan; ^2^Department of Gastroenterology, Ishinkai Yao General Hospital, 1-4 Numa, Yao-city, Oska 581-0036, Japan; ^3^Department of Gastroenterology & Hepatology, National Center for Global Health and Medicine, 1-21-1 Toyama, Shinjyuku-ku, Tokyo 162-8655, Japan

## Abstract

This study aimed to assess and compare effectiveness of Autofluorescence imaging (AFI) in diagnosis of early gastric cancer (EGC) between experienced and less experienced endoscopists. Fifty selected images (20 neoplastic lesions and 30 benign lesions/areas) of both white light endoscopy (WLE) and AFI were blindly reviewed by two groups; first consisted of five experienced endoscopists and second included five less experienced endoscopists. Sensitivity, specificity, and accuracy were 70%, 78%, and 75%, respectively, for AFI and 81%, 76%, and 78%, respectively, for WLE in the experienced group. In the less experienced group, sensitivity, specificity and accuracy were 80%, 81% and 80%, respectively, for AFI and 65%, 77%, and 72%, respectively, for WLE. Interobserver variability for the less experienced group was better with AFI than WLE. AFI improved sensitivity of endoscopic diagnosis of neoplastic lesions by less experienced endoscopists, and its use could beneficially enhance the clinical effectiveness of EGC screening.

## 1. Introduction

Gastric cancer incidence and mortality have declined dramatically over the past 70 years [1]. Despite these declines, gastric cancer is still the fourth most common cancer and the second leading cause of cancer-related deaths worldwide [2]. Development of esophagogastroduodenoscopy (EGD), a screening tool for early gastric cancer (EGC), in place of radiology [3] has allowed widespread availability of screening in high-risk countries such as Japan and Korea resulting in decreased mortality. In contrast, relatively few gastric cancers are discovered at an early stage in most Western countries [4].

We have witnessed firsthand significant advances in endoscopic treatment for early gastric cancer in recent years including development of endoscopic submucosal dissection (ESD) [5–7]. In order to fully benefit from the advantages of endoscopic treatment, however, it is important to detect gastric cancers at the earliest possible stage [8]. Most cases of EGC are slightly depressed or elevated lesions and red or pale in color, but some EGC are quite flat and almost isochromatic so there is very little contrast with the surrounding mucosa. Such subtle changes of EGC can make for a challenging endoscopic diagnosis. The difficulties involved in making an accurate diagnosis can be compounded by the inexperience of some endoscopists particularly in countries where the incidence of gastric cancer is low.

Following development of a fluorescence detection method for neoplastic lesions in 1957, autofluorescence imaging (AFI) has attracted considerable attention in the diagnosis of early cancerous lesions [9, 10]. AFI is a novel imaging method that produces computerized real-time pseudocolor images by detecting faint fluorescence emitted from endogenous fluorophores exposed to excitation light. Neoplastic lesions with an altered fluorescence can be distinguished from the enhanced surrounding normal pattern by variations in color.

Several published reports have examined the advantages of AFI for detection of colorectal cancer [11–14]. It may also be easier for less experienced endoscopists to detect gastric neoplastic lesions using AFI even when such lesions cannot be detected by conventional white light endoscopy (WLE) [15]. The aim of this pilot study was to assess and then compare the effectiveness of AFI in the diagnosis of gastric neoplastic lesions between experienced and less experienced endoscopists.

## 2. Methods

### 2.1. Study Design

During endoscopy using a prototype AFI system that included both WLE and AFI functions performed by one experienced endoscopist (C. Yokoi), pictures of neoplastic lesions and benign lesions/areas were taken from 44 patients with EGC after obtaining their informed consent who were referred to our hospital for treatment from August 2005 to March 2006. Pictures of 45 EGCs were collected along with 172 pictures of benign lesions/areas from these 44 patients. All neoplastic and benign lesions were assessed histopathologically from biopsy specimens. Pictures of poor quality were excluded, and 50 pictures were then selected at random by the study coordinator (K. Tada) for this pilot study including 20 pictures of neoplastic lesions (four adenomas and 16 EGCs) and 30 pictures of benign lesions/areas (four polyps, six ulcer scars, four atrophic changes, and 16 normal mucosal areas). The clinicopathological characteristics of the neoplastic lesions were classified based on the Japanese Classification of Gastric Carcinoma [16] while the descriptions of WLE and AFI colors were determined by the study coordinator (Table 1). All slightly elevated and flat lesions appeared magenta in a green field, and 7 of 9 slightly depressed lesions displayed green in a magenta field. The mean lesion size was 20 mm. We prepared 50 sets of AFI and WLE images for the same selected lesions and normal mucosa. Each image was assigned a random sequence number with the 50 AFI images displayed first followed by the 50 WLE images. A review of the images was performed individually by 10 endoscopists excluding the endoscopist who took the images and the study coordinator who were divided into two separate groups: five endoscopists with extensive experience in EGC from the National Cancer Center Hospital (NCCH) and five less experienced endoscopists working in a general hospital. Each of the endoscopists in the first group of reviewers had over 10 years of medical experience including more than three years at NCCH and had evaluated in excess of 700 EGCs annually. The endoscopists in the second group of reviewers each had less than five years of medical experience and had evaluated fewer than 30 cases of EGC per year. No information regarding any of the lesions was available to the reviewers. An answer sheet was given to each endoscopist with two options regarding each image: “neoplasm exists” or “no neoplasm.”

### 2.2. Autofluorescence Imaging System

The prototype AFI system used in this study (XGIF-Q240FZ; Olympus Medical Systems Corp., Tokyo, Japan) was equipped with two charge-coupled devices (CCDs) at the tip of the endoscope that could easily be switched by pushing a single button on the scope handle: one for high-resolution white-light observation and the other for autofluorescence observation. The AFI system digitally creates real-time pseudocolor images from autofluorescence (excitation at 390–470 nm and detection at 500–630 nm) and green reflection (G′) at 540–560 nm. The system relies on a sequential method in order to provide clear image profiles and distinguish autofluorescence reduction of neoplastic lesions caused by hemoglobin absorption.

### 2.3. AFI Diagnostic Criteria for Neoplastic Lesions

**Figure 1:**
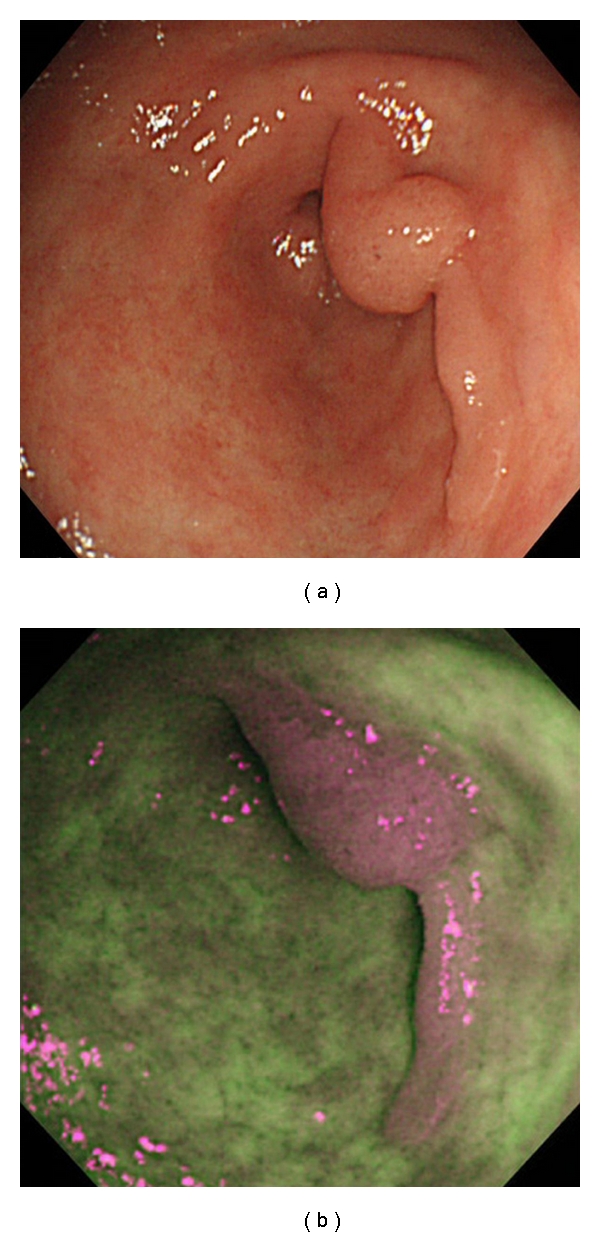
Diagnostic criteria for autofluorescence imaging (AFI). We defined a lesion suspected of being neoplasia using AFI (AFI-positive) as an area that was clearly different from the surrounding mucosa in color. (a) WLE image of an EGC. (b) AFI-positive image displayed the same EGC as a magenta area with defined margins within the green-colored mucosa.

A neoplastic lesion was defined for AFI purposes as an area that contrasts in color with the surrounding background such as “a magenta area in a green field” or “a green area in a magenta field” (Figure 1).

AFI images are considerably different from those of conventional WLE, however, so endoscopists have to become familiar with such images in order to attain an appropriate level of diagnostic skill. All participating endoscopists in this study were briefed on how to evaluate AFI images and given an opportunity to review 10 sample pictures beforehand at a 30-minute training lecture.

### 2.4. Statistical Analysis

We compiled the answers for the five endoscopists in each group and then calculated sensitivity, specificity, and accuracy for both groups. Data were analyzed using the chi-square test, and value differences of *P* < 0.05 were considered statistically significant. Interobserver variability was determined for each group using Kappa (*κ*) statistics. All statistical analyses were performed using STATA version 10.0 (StataCorp, College Station, Tex, USA).

## 3. Results

Detection of neoplastic lesions by the experienced endoscopists using AFI and WLE, respectively, resulted in a sensitivity of 70% (95% CI 60–78%) and 81% (95% CI 72–88%), a specificity of 78% (95% CI 71–84%) and 76% (95% CI 69–82%), and an accuracy of 75% and 78%. Less experienced endoscopists had a sensitivity of 80% (95% CI 71–87%) and 65% (95% CI 55–74%), a specificity of 81% (95% CI 74–86%) and 77% (95% CI 70–83%), and an accuracy of 80% and 72%, respectively, using AFI and WLE for diagnosis. Sensitivity in the less experienced group of endoscopists using AFI (80%) was significantly higher than when using WLE (65%) (*P* < 0.05). And sensitivity in the less experienced group of endoscopists using AFI (80%) was comparable to the more experienced group of endoscopists using WLE (81%) (Figure 4). 

Interobserver variability for detection of neoplastic lesions by the group of less experienced endoscopists was better for AFI than with WLE (experienced group: AFI [*κ* = 0.42 (95% CI 0.33–0.51)] and WLE [*κ* = 0.52 (95% CI 0.43–0.61)]; less experienced group: AFI [*κ* = 0.52 (95% CI 0.43–0.61)] and WLE [*κ* = 0.29 (95% CI 0.20–0.38)]). There was no statistically significant difference in the interobserver variability using AFI between the experienced and less experienced endoscopist groups. In contrast, there was a significant difference using WLE between the two groups with the experienced endoscopist group having significantly better interobserver variability (Table 2). 

**Figure 2:**
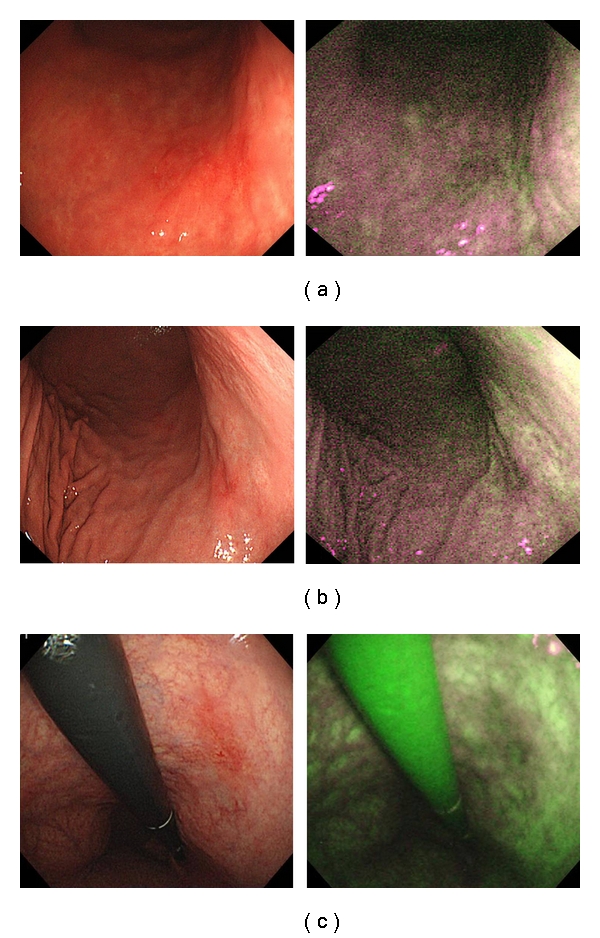
These three neoplastic lesions were diagnosed more easily using WLE. All three appeared reddish in color with a slightly depressed area.

**Figure 3:**
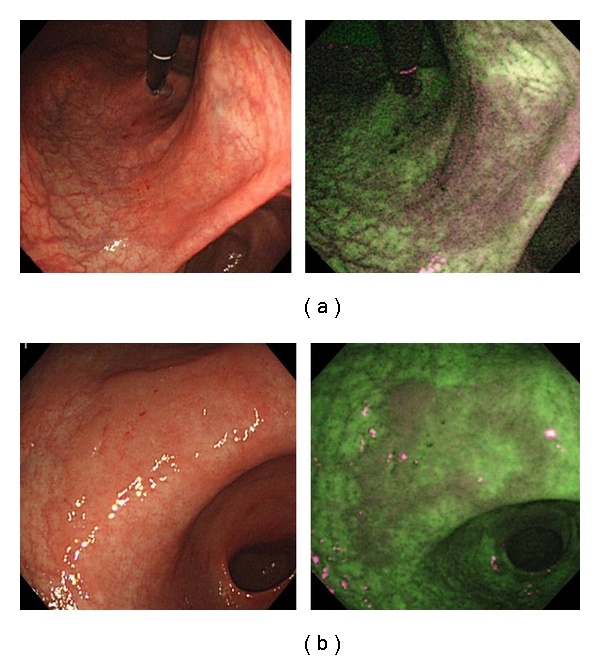
Here are two examples of neoplastic lesions diagnosed more easily using AFI. Each of them appeared as an isochromatic flat lesion using WLE.

**Figure 4:**
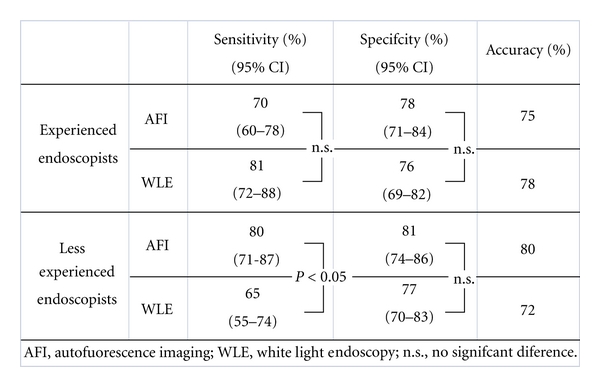
AFI and WLE image review results.

With regard to lesions diagnosed by the group of less experienced endoscopists, three of the 20 (15%) neoplastic lesions were diagnosed more often by WLE, and 11 (55%) were diagnosed more often by AFI. All three (100%) neoplasias diagnosed more often by WLE were slightly depressed lesions. (Figures 2(a), 2(b), and 2(c)). In contrast, eight of the 11 (73%) neoplasias diagnosed more often by AFI were flat lesions (Figures 3(a) and 3(b)). 

## 4. Discussion

The effectiveness of AFI for diagnosing EGC by highly experienced endoscopists has been assessed in several studies, but there are no published reports evaluating less experienced endoscopists [15, 17]. 

AFI can differentiate tissue types based on variations in their fluorescence emissions. When tissue is exposed to short wavelength (390–470 nm) light, endogenous biological substances such as collagen, nicotinamide adenine dinucleotide, flavin, and porphyrins are excited leading to the emission of longer wavelength (500–630 nm) fluorescent light (autofluorescence) [18]. Neoplastic and nonneoplastic tissues have different autofluorescence characteristics including nuclear-cytoplasmic ratio, mucosal layer thickness, and volume of blood flow [19]. These characteristics may facilitate differentiating between the two. During endoscopy using the AFI mode, neoplastic lesions contrast with normal mucosal tissue (i.e., “a magenta area in a green field” or “a green area in a magenta field”).

A number of studies have reported that AFI is effective for colorectal cancer screening, but this is still debatable while its suitability for gastric cancer screening remains somewhat more controversial [11–14, 20, 21]. Inflammatory and hyperplastic changes in the stomach can alter mucosal layer thickness and blood flow volume causing autofluorescence contrast variations with similar appearance to neoplastic lesions. Such difficulties are also reported in Barrett's esophagus [22]. False-positive results and low specificity, therefore, are more common in the stomach and Barrett's esophagus. Currently, AFI cannot distinguish precisely between gastric neoplastic lesions and inflammatory or hyperplastic changes. It is already known, however, that EGC is not easily detected by less experienced endoscopists. No detection, of course, means there is no treatment, so our primary objective in EGC screening should be higher sensitivity rather than diagnostic accuracy. False-positive lesion findings should be a secondary consideration to the actual sensitivity rate. AFI provides a simple dichromatic difference that may help less experienced endoscopists diagnose neoplastic lesions more easily. For this reason, we included less experienced endoscopists as well as highly experienced endoscopists in our study.

In the group of experienced endoscopists, the WLE sensitivity of 81% was reduced to 70% with AFI although there was no statistically significant difference indicating that AFI did not provide an advantage in terms of detection for that particular group. We postulate that sensitivity using WLE was already high in the experienced endoscopists group as variables such as surface irregularity, elasticity, thickness, hardness, converging folds, and background status were examined. The ability to interpret those changes using WLE improves with endoscopic experience. We believe that experienced endoscopists in this study attempted to interpret all characteristics of a lesion using AFI rather than just color contrast. Reliance on such variables, in fact, can mislead experienced endoscopists given AFI's low vision quality.

In contrast, AFI raised detection sensitivity from 65% to 80% and interobserver variability from 0.29 to 0.52 for less experienced endoscopists. Although the subtle mucosal changes of EGC make endoscopic diagnosis a challenge for less experienced endoscopists using WLE, our findings indicated that AFI might facilitate easier diagnosis of neoplastic lesions by such endoscopists. This was likely due to objective evidence of a definite difference in coloration between neoplastic lesions and the surrounding mucosa. AFI was particularly effective in the diagnosis of flat lesions. The overall sensitivity and interobserver agreement were unsatisfactory, however, for the differential diagnosis between neoplastic and benign lesions so we still need to perform a biopsy.

There are, however, a number of limitations to this pilot study. Firstly, we used still images taken by experienced endoscopists, and some of those lesions may not have been detected at all by less experienced endoscopists during real-time endoscopy. Quality of the AFI view depends on technical skill so less experienced endoscopists might not be able to reproduce the images used in this study. Our results, therefore, may not be reflected in actual examination, but the results of less experienced endoscopists were in fact better than experienced endoscopists using the same AFI pictures. In the future, effectiveness of AFI for screening of EGC should be assessed in a prospective study including experienced and less experienced endoscopists with diagnosis on a real-time basis. Secondly, in order to make it simpler, we included only two options “neoplasm exists” or “no neoplasm” for reviewers. It would have been better to also have them evaluate lesion characteristics such as AFI and WLE colors as well as macroscopic type. So we plan to conduct the real-time evaluations lesion features in the next study. Thirdly, there was no yardstick used in choosing the specific kinds and relative percentages of images presented in this study, and the percentage of neoplastic lesions was considerably higher than than that which would normally be the case in routine gastric screening. The actual choice of images could have had an effect on the results. For example, Kato et al. carried out a prospective study on the effectiveness of AFI for detecting EGC [17]. They reported sensitivity of 74% and specificity of 83% for WLE and sensitivity of 64% and specificity of 40% for AFI performed by experienced endoscopists. Data for the experienced endoscopists in our study showed a similar results regarding sensitivity of AFI. Although the high specificity of 78% with AFI in our study may have been affected by the choice of images, the sensitivity results in both groups of endoscopists were quite promising.

A number of practical improvements need to be made before AFI can actually be introduced into a clinical gastric screening setting (i.e., the AFI system video endoscope is too large in diameter with poor flexibility and lower overall image quality), but we believe that AFI has the potential to increase the sensitivity of endoscopic diagnosis of neoplastic lesions by less experienced endoscopists. This would be important not only in Japan but especially in those countries with a low incidence of gastric cancer. The AFI system is only being used on a limited basis in Japan and a few other countries at the present time, and greater availability and increased usage worldwide of this system should demonstrate its effectiveness and lead to wider acceptance.

The primary advantage of AFI is that it identifies suspicious lesions as areas evidencing color contrast almost instantaneously throughout the entire endoscopic field. Even if the false-positive rate using AFI is high, the examining endoscopists can use other modalities such as chromoendoscopy or NBI with magnification in addition to obtaining biopsies to verify their initial suspicion of EGC [23, 24]. This is provided, of course, that lesions are detected in the first place. AFI could then become an important technique for EGC screening by all endoscopists to diagnose suspected lesions.

This is the first study on the effectiveness of AFI by less experienced endoscopists. Although the results are encouraging, it should be noted that this was an uncontrolled pilot trial involving a relatively small number of lesions. Prospective randomized controlled trials involving a large number of subjects would be beneficial in the future to more fully evaluate the effectiveness of AFI in the diagnosis of EGC.

In conclusion, the use of AFI in this study increased sensitivity in the endoscopic diagnosis of gastric neoplastic lesions by less experienced endoscopists. Such use may beneficially enhance the clinical impact of EGC screening by less experienced endoscopists, but this will need to be confirmed in a prospective study with diagnosis on a real-time basis.

## Figures and Tables

**Table 1:** Neoplastic lesion characteristics and AFI colors.

		Number of lesions	AFI color	
			Magenta	Green
Pathological type	Carcinoma (differentiated)	13	9	4
	Carcinoma (undifferentiated)	3	0	3
	Adenoma	4	4	0

Location	Upper third of stomach	2	1	1
	Middle third of stomach	9	6	3
	Lower Third of Stomach	9	6	3

Macroscopic type	Elevated	9	9	0
	Flat	2	2	0
	Depressed	9	2	7

WLE color	Reddish	9	4	5
	Isochromatic	8	8	0
	Pale	3	1	2

AFI: autofluorescence imaging; WLE: white light endoscopy.

**Table 2:** Interobserver variability for detection of neoplastic lesions with AFI and WL.

*κ*	AFI (95% CI)	WLE (95% Cl)
Experienced endoscopists	0.42 (0.33–0.51)	0.52 (0.43–0.61)
Less experienced endoscopists	0.52 (0.43–0.61)	0.29 (0.20–0.38)

AFI: autofluorescence imaging; WLE: white light endoscopy.
